# Embedded MicroHeating Elements in Polymeric MicroChannels for Temperature Control and Fluid Flow Sensing

**DOI:** 10.6028/jres.109.025

**Published:** 2004-06-01

**Authors:** Michael Gaitan, Laurie E. Locascio

**Affiliations:** National Institute of Standards and Technology, Gaithersburg, MD 20899-8124

**Keywords:** flow sensor, microfluidics, microheating elements, polymer microchannels

## Abstract

This paper describes the first demonstration of temperature control and flow sensing of fluids using integrated circuit (IC)-based microheating elements embedded in microchannels molded in polydimethylsiloxane (PDMS). Fluid channels and connections to capillary tubing are molded in PDMS using a silicon wafer template. The PDMS film is then bonded to an IC that contains the micromachined microheating elements. Capillary tubes are inserted and fluids are externally pumped through the channels. Heating of the fluid is observed by the formation of bubbles on the microheating element. Sensing of fluid flow is demonstrated by measuring a change in the large signal resistance of the microheater analogous to a hot wire anemometer with a detection limit of ± 320 pL/s.

## 1. Introduction

Over the past several years there has been substantial work in the development of the fluidic components for micro-total analytical system (µTAS) and biochip technologies. These components include separation channels [1,2,3,4], polymerase chain reaction (PCR) [5,6], monolithic micro-columns [7], electro-osmotic pumps [8], external pressure driven pumps, bubble pumps [9], valves, and fluidic interconnects and interfaces [10].

These fluidic components have been realized in a variety of substrate materials including silicon, glass, and polymers. A number of trade-offs have been recognized to exist between the choices of substrate materials. For example, silicon/glass systems have the advantages of well-understood surface chemistries and compatibility with IC fabrication processes and electronics. In contrast, plastics offer advantages in cost reduction and ease of fabrication but do not have a clear path towards the integration of electronic and active components.

Approaches for integration of fluidic components have been pursued by a hybrid-package [10] approach and by direct integration in an IC compatible process [11,12]. Both of these methods require the use of silicon as a substrate material.

Integration of thermal elements in microfluidic systems has important applications including the control of chemical reaction rates, temperature cycling, and fluid flow measurement and control. Recently, a pulsed mode thermal fluid flow sensor for microfluidic systems was reported [13]. The sensor was fabricated by a silicon micromachining process and then anodically bonded to a glass plate for sealing and for providing inlet and outlet fluid ports. Pulsed mode operation of the device was used to reduce the sensitivity of the sensor to fluctuations in ambient temperature.

Our work has been focused on the development of fabrication methods for integration of active components on plastic substrate materials by pick-and-place tools. We call this approach to integration drop-in functionality. Using *drop-in functionality*, a plastic substrate can be patterned with some network of fluid channels and other passive fluidic elements. Bonding to a top plastic plate that contains a fluid network and fluid interconnects would then provide a seal to the plastic substrate. Silicon/glass/IC-based active elements (with appropriate fluidic and electrical interface) are embedded in the locations to perform the desired functions, e.g., sample manipulation and detection. The advantages of this approach are that active elements important for control and detection of chemical reaction can be integrated in the locations where their functionality is required while still preserving the advantages of using plastic materials for the passive fluidic elements.

In this paper, we present results of integration of microheating elements fabricated on an integrated circuit chip with fluid microchannels in a molded polydimethylsiloxane (PDMS). The PDMS film is cut into a small rectangular chip and positioned over the integrated circuit chip containing a microheating element. The two chips are aligned and bonded so that the microchannel is directly over the microheater. The microchannel is interfaced to an external pump and capillary tubing using a novel method that we have developed to create a capillary interface using commercially available fittings for the mold.

Results for the operation of the assembled device are presented. Fluid flow rate is measured by observing the time of flight of particles in the channel using a florescence microscope. Fluid heating by the microheating element is observed by the formation of steam bubbles in the channel. Finally, the microheating element is demonstrated as a thermal fluid flow sensor with operation analogous to a hot wire anemometer.

## 2. Experimental Method

We present a fabrication method for the integration of the silicon IC-based microreactive elements with molded fluid channels. The fluid channels are molded [4] using a micromachined silicon wafer template [14] as a cast. Ferules are attached to the template to create a mold for an interface to capillary tubing. The IC chips containing the microheating elements are prepared and then bonded to the fluid channels. The steps to fabricate and assemble the microreactive elements will be described in this section, beginning with the fabrication of the silicon template.

### 2.1 Fabrication of Silicon Templates

We first reported use of silicon templates for imprinting channels in plastic in a previous paper [14]. The process sequence for making the silicon templates will be described in detail in this paper. Low-doped (100) silicon wafers are placed in a wet oxidation furnace at 980 °C for 20 min to create a 100 nm thick oxide film^1^. Following this, photoresist is spun-on the wafer and the wafer is soft baked. Hexamethyldisilazane is spun-on before application of the photoresist as an adhesion promoter. In addition, photoresist is spun-on to the backside of the wafer. Finally, the wafers are rinsed in deionized (DI) water and spin dried.

A photomask is prepared for patterning the photoresist. For this work, our designs require a 20 µm minimum feature size. We create the photomasks by high-resolution printout onto clear transparency film. The mask contains the drawings of the fluid channel elements and an alignment mark for rotational alignment to the flat of the silicon wafer. All features are drawn in manhattan format, i.e., lines or boxes parallel or perpendicular to the alignment mark, so that they will be aligned to the {111} planes of the silicon wafer. This alignment is required for anisotropic etching of the silicon wafer. The mask is placed over the front side of the silicon wafer and aligned to the flat using the alignment mark. The photoresist is exposed using a UV light source and patterned in developer, then rinsed and dried. Once the photoresist is patterned, it is used to pattern the silicon dioxide film by a 2 min wet etch in a 6 % buffered HF oxide etch solution, then rinsed and dried. Following this, the photoresist is stripped in acetone and then the wafer is again rinsed and dried.

At this point, the wafers are ready for the anisotropic etch in tetramethylammonium hydroxide (TMAH) solution. We prepare a TMAH etch solution, based on the work reported by Tabata et al. [15], consisting of 450 mL of 25 % by mass electronic grade TMAH in aqueous solution mixed with 900 mL of DI water (1 part 25 % TMAH to 2 parts DI water). The solution is placed in a reflux etch container and heated to 80 °C on a hotplate. The wafers are placed in a Teflon holder and immersed in the solution. Following the work by Klassen et al. [16], 1 g of ammonium peroxydisulfate (APODS) in powder form is added every 10 min during etching in order to eliminate the formation of hillocks.^2^ The etch rate for this solution in the <100> direction was measured as 0.9 µm/min.

Finally, the silicon dioxide mask is stripped by a second 2 min wet etch in 6 % buffered HF, then rinsed and dried. The cross section of the template a trapezoidal-shaped line with a width at the top surface of 20 µm. The sides of the trapezoid are aligned to the {111} planes of the silicon crystal forming a 54.74 °C angle from the wafer surface plane [17].

### 2.2 Casting PDMS With Capillary Interconnects

The silicon templates were used to mold PDMS films following similar procedures described by Effenhauser [18] and Duffy [4]; however, we developed a method to form an interface for capillary tubing using commercial off-the-shelf capillary tubing hardware. Commercially available capillary tubing, tubing sleeves, and fittings were used to create the fluid interconnects. The tip of a capillary tube fitting was cut and glued (using a quick setting epoxy) to the template at each end of the microchannels for the fluid connection. Teflon sleeves were then inserted into fittings.

**Fig. 1:**
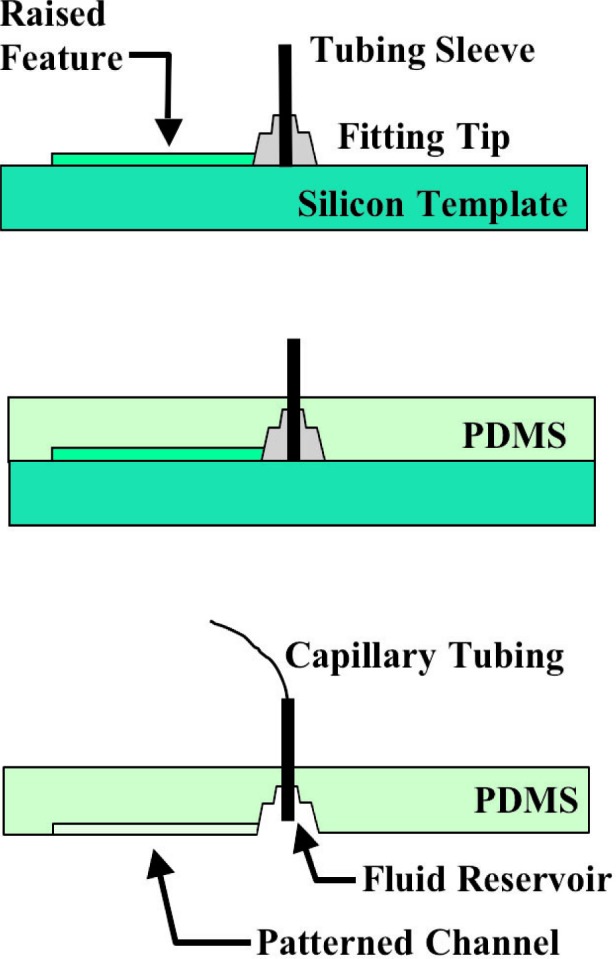
Schematic of process used to mold the PDMS film with capillary connections.

Figure 1 shows a schematic of the process used to mold the PDMS film. The silicon template with the attached fittings was placed in a dish. The uncured polymer was poured over the template to a thickness of approximately 0.5 cm and cured at room temperature for 24 h. After curing, the polymer membrane was cut using a scalpel into discrete PDMS chips that would later be bonded to the ICs containing the microheating elements. The PDMS chips were individually peeled away from the silicon template using tweezers. As the chips were peeled away, the fitting tips remained bonded to the silicon template creating a fluid reservoir while the tubing sleeves remained embedded in the membrane. The PDMS chips incorporated a microchannel configuration with two fluid reservoirs, which is designed for alignment to the microheating elements. Capillary tubing could then later be inserted into the tubing sleeves to interface the external pump to the channel.

### 2.3 MicroHeating Element Fabrication

The IC-based microheating elements that were used in this work have been previously developed, studied, and described extensively for applications such as thermal radiation elements [19], thermal flat panel displays [20], and microhotplate gas sensors [21] at NIST. The microheating elements were operated in air and vacuum environments. They have been shown to have a linear relationship with operating temperature and power dissipation of 40 °C/mW throughout their operating temperature of ambient to 1000 °C. It has also been reported that they can be controlled in a pulsed temperature mode with millisecond time constants [22] throughout their operating temperature.

The fabrication process for the microheating elements is briefly described in this paragraph. The microheating elements were designed using IC layout software that was modified to include an *open* tile [23]. The ICs were fabricated through a commercial IC foundry through the MOSIS Service [24]. The ICs received from the foundry contained precursors of the microheating element that contained openings in the SiO_2_ passivation film created by the layout of the *open* tiles. To complete their fabrication, the microheating element precursors were silicon micromachined by wet anisotropic silicon etching in TMAH [25] in order to mechanically release them from the silicon substrate without dissolving the aluminum bond pads.

**Fig. 2:**
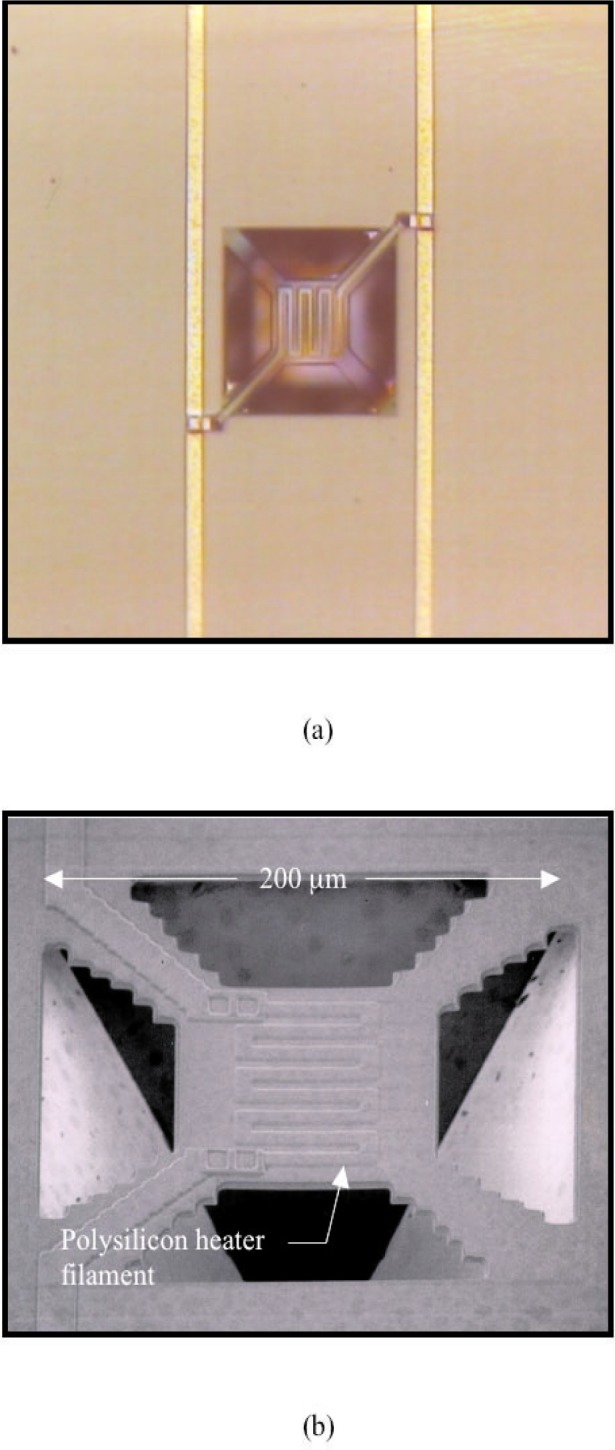
Optical micrograph (a) and scanning electron micrograph (b) of two similar fully fabricated microheating element.

Figure 2 shows an optical micrograph and a scanning electron micrograph of a fully fabricated microheating element. The microheating element was designed to be 200 µm square in size. The element consists of a trampoline-shaped membrane of glass that is suspended over a trapezoidal shaped pit in the silicon substrate. The “tethers” at the four corners of the trampoline mechanically support the square plate in the center. The plate in the center contains a polysilicon resistive filament that is encapsulated in the glass membrane. Two of the tethers contain electrical connections from the filament to external bond pads on the IC. The sides of the trapezoidal pit are aligned with the {111} planes of the silicon substrate. The openings in the SiO_2_ passivation film are determined by the layout of the *open* tiles in the IC design. Following the fabrication process, the IC chip containing the microheating element was attached and wire bonded in a standard 40-pin dual-in-line package (DIP).

### 2.4 Assembly

The PDMS chip containing the fluid microchannel was bonded and sealed to the IC chip containing the microheating elements using an oxidation process described by Schueller et al. [26]. After packaging, the IC was placed in an oxygen plasma reactor at 26.7 Pa (200 mTorr) for 5 min. Following this, the PDMS chip was placed with the IC chip in oxygen plasma for 5 s. It was found that longer treatments of the PDMS chip in the oxygen plasma hardened and discolored it and also resulted in weak bonds.

**Fig. 3:**
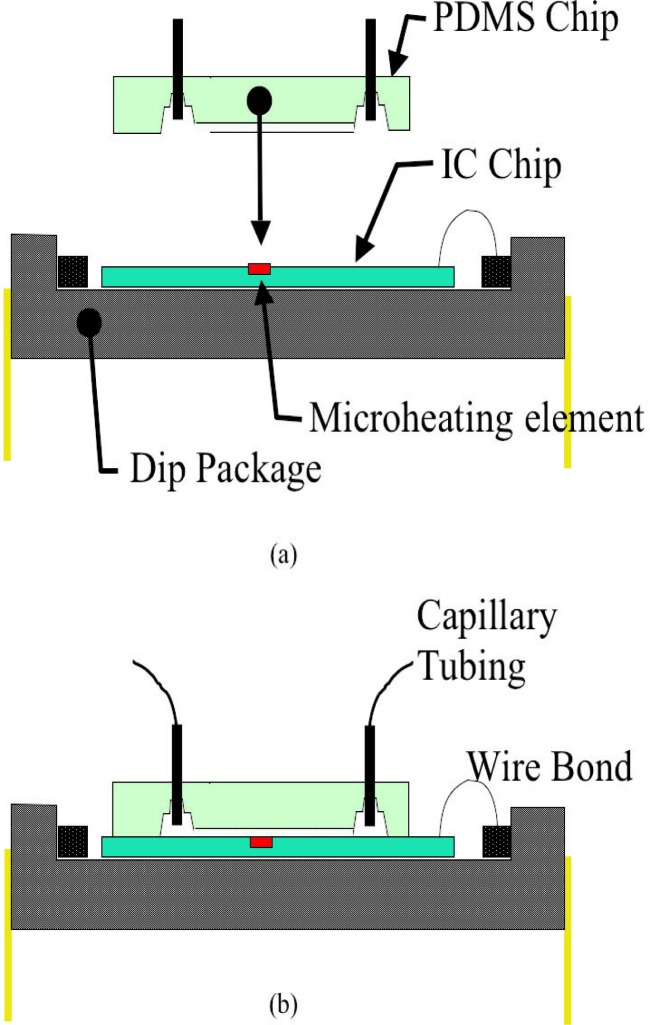
Cross-sectional diagram of the assembly process. The PDMS chip is positioned over the packaged IC chip (a) and brought into contact (b). The device is left undisturbed overnight. Capillary tubes are inserted into the tubing sleeves and connected to an external pump.

**Fig. 4:**
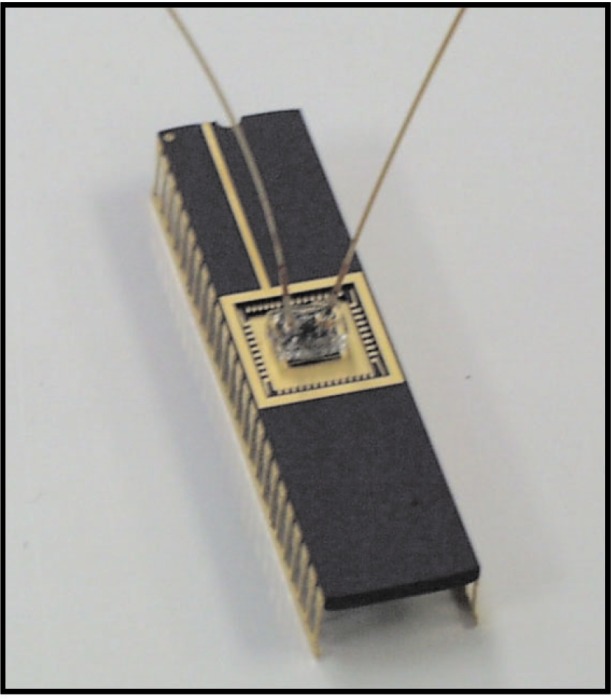
Photograph of the fully assembled device with capillary tubes inserted into the tube sleeves.

Figure 3 shows a diagram of the assembly process. After exposure to the plasma, the IC chip and the PDMS chip were hand assembled under a stereo optical microscope using tweezers to position the microchannel patterned in the PDMS chip over the microheating element. The devices were left undisturbed overnight before handling. It was found that leaving the chips undisturbed overnight resulted in strong bonds between the PDMS and the SiO_2_ surface of the IC chip. Figure 4 is a photograph of the fully assembled device with capillary tubing inserted into the tube sleeves.

The experimental setup for this study was designed as follows: the two capillary tubes that were inserted into the capillary interface tubing sleeves shown in Fig. 4 were used to vacuum pump fluid from a reservoir through the microchannel. One tube was run into a fluid reservoir that was filled with deionized and degassed water. The second capillary tube was run to the vacuum pump. The vacuum had a pressure indicator display.

### 2.5 Liposome Preparation

Liposomes were prepared by the injection method described [27] in previous publications and encapsulated 100 mmol L^–1^ of the fluorescent dye, 5-(and –6) carboxyfluorescein (CF) (Molecular Probes, Eugene OR).^3^

## 3. Results and Discussion

In this section, we present the measurement results for integrated heating elements in molded fluid channels. The pumping of fluids through the capillary interface was first tested. Following this, the temperature of the fluid was thermally cycled using the embedded heating elements. Finally, the microheating elements were used to measure fluid flow.

### 3.1 Observation of Fluid Flow

In this experiment, liposomes that encapsulated a fluorescent dye [28] were mixed to form a colloidal suspension with the DI water in the fluid reservoir. Fluid was vacuum pumped from the reservoir into the microchannel.

**Fig. 5:**
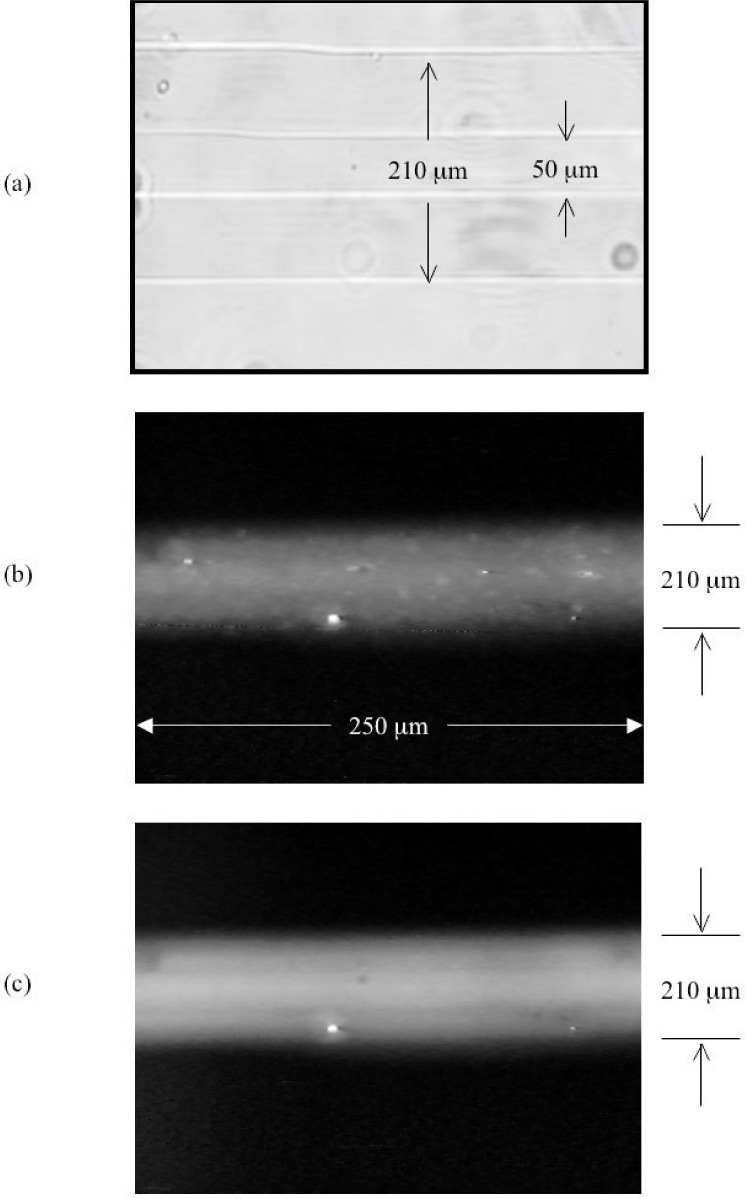
Optical micrograph (a) of the microchannel in PDMS with trapezoidal cross-sectional dimensions of 50 µm at the top and 210 µm at the bottom of the channel. Florescence micrographs of fluid flow in the microchannel with fluorescent particles in the fluid: (b) with a time of flight measured velocity of 250 µm/s and (c) much faster.

Figure 5a is an optical micrograph of the fluid channel in the PDMS taken looking down on the device. The cross sectional area of the fluid channel is in the shape of a trapezoid corresponding to the trapezoidal shape of the lines created on the silicon template. The channel had a dimension at the top of the trapezoid of 50 µm and 210 µm at the bottom. This corresponded to a 110 µm channel depth and a 9500 µm^2^ cross-sectional area.

Figure 5b is an optical florescence micrograph of the liposomes encapsulating florescent dye flowing through the channel with a vacuum pressure of 5 cm Hg measured at the pump. The particles moving through the channel traversed the 250 µm distance indicated in the figure in 1 s corresponding to a particle velocity of 250 µm/s. It was noted that particles close to the walls of the channel appeared to move at a slightly slower velocity than particles in the center of the channel; however, assuming a constant flow velocity, the flow rate was calculated to be 2.4 nL/s.

Figure 5c is an optical florescence micrograph of the liposomes flowing through the channel at a higher rate corresponding with a vacuum pressure of 20 cm Hg measured at the pump. In this case, the particle flow velocity was too fast for visual measurement of fluid flow rate.

### 3.2 Thermal Cycling of Fluids

The microheating element was thermally cycled in a stagnant liquid, i.e., no fluid flow in the channel, by switching on and off a current source that was electrically connected to the filament. The on-state current was increased to a point where bubbles could be observed forming to the heater.

**Fig. 6:**
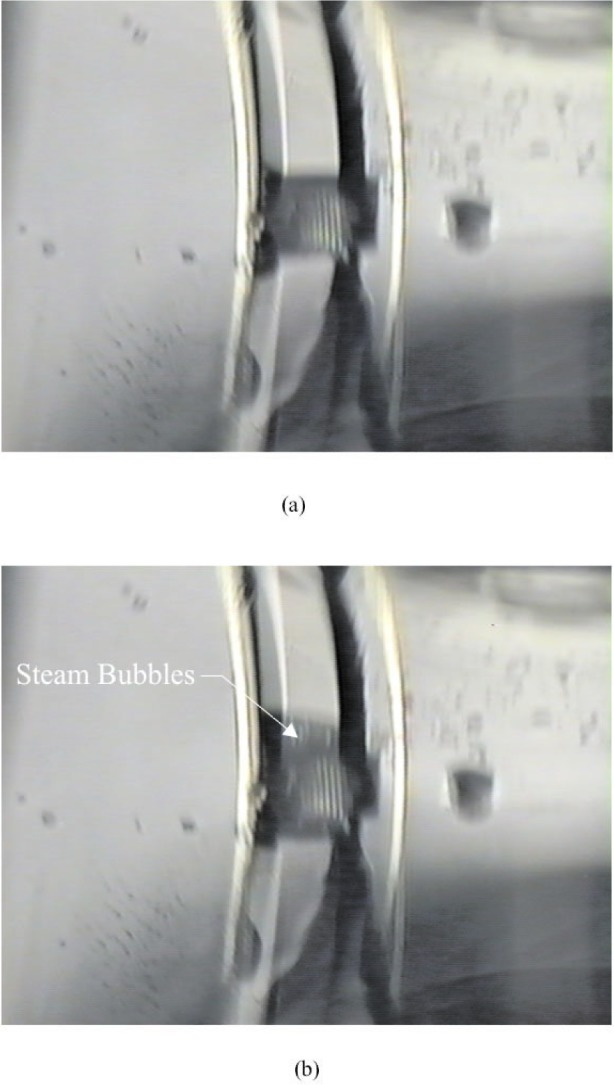
Microheater operation: (a) heater is off and, (b) heater is on creating steam bubbles.

Figure 6 is an optical micrograph of the microheating element in the fluid channel. Figure 6a corresponds to the heater off-state (no current flow). Figure 6b corresponds to the heater operated in the on-state with a current flow of 10 mA.

It was noted that many bubbles (as opposed to one bubble) formed above the microheating element and were much smaller in size in comparison to the device. This indicated that many nucleation sites were available for the bubbles to form on the heating element. This might be attributed to the geometry of the device since it contained a cavity beneath the membrane with openings that could pass liquid, as shown in Fig. 2b.

By visual observation, the bubbles would dissipate instantaneously when the power was switched off to the microheating element. This indicated that the liquid was in a sub-critical boiling condition.

### 3.3 Thermal Fluid Flow Sensor

The electrical resistance of the microheating element was measured to be 3.2 kΩ at low current, e.g., negligible heating of the filament so that it was approximately at ambient temperature. As the power dissipation of the filament is increased, its temperature correspondingly increases causing an increase in the electrical resistance. This effect is characterized by a linear temperature coefficient of resistance. The temperature coefficient of resistance (*TCR*) of the polysilicon filament of microheating elements fabricated using this approach has been previously investigated and characterized to be 0.001/°C. The electrical resistance of the microheating elements follow the linear relationship of

$$R=R_{0}[1+TCR(T-T_{0})],$$ (1)

where *R*_0_ is the resistance at low power dissipation at temperature *T*_0_, and *T* is the temperature of the polysilicon filament resulting in a resistance *R*.

The relationship between the temperature of the filament and the power dissipation is related to the thermal conductivity of its surrounding medium. If the surrounding medium is composed of a fluid (or gas) that is flowing over the heating element, a steady state equilibrium condition will be established between the temperature of the filament and the power dissipation. If the flow rate of the liquid is changed, a new equilibrium temperature will be established.

**Fig. 7:**
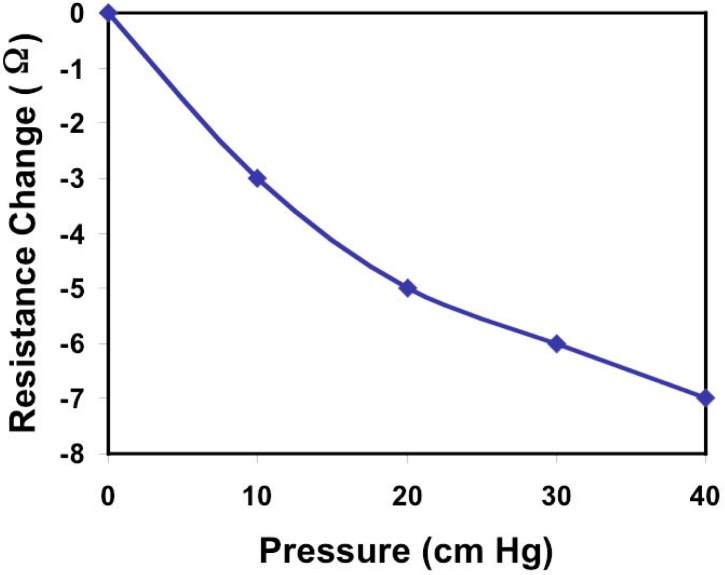
Fluid flow effect on resistance of the microheater. The vacuum pressure of the pump connected to the capillary tubing is plotted on the *x*-axis, which corresponds to the fluid flow rate in the microchannel. The resistance change of the microheating element due to thermal coupling of the heater with the fluid is plotted on the *y*-axis. The resistance of the heater decreases as the pressure is increased (fluid flow rate increases) analogous to a hot wire anemometer.

The change in heater temperature from the first state of equilibrium to the second will result in a corresponding change in the electrical resistance on the microheater according to Eq. (1). This change in resistance, Δ*R* = *R*_0_ – *R*, is plotted as a function of pump pressure in Fig. 7.

In this measurement, the power dissipation of the heater was adjusted to give rise to a 3.3 kΩ electrical resistance. Using Eq. (1), this corresponds to an approximate^4^ 31 °C temperature rise of the microheating element from ambient (room) temperature. A high precision current-voltage meter was used to measure the resistance of the heater. In this measurement, the fluctuation of the resistance was on the order of ± 0.2 Ω.

The resistance change in Fig. 7 is plotted with respect to zero fluid flow, i.e., *R*_0_ corresponds to no flow. As the flow rate is increased, the resistance changes as plotted in the figure to a maximum difference of 7 Ω at a pump pressure of 40 cm Hg. This corresponds to a fractional resistance change on the order of 0.2 % at 40 cm Hg.

The observation of particle velocity in the preceding section yielded a 2.4 nL/s flow rate at 5 cm Hg. The resistance change of the microheating element at this corresponding pressure is on the order of 1.5 Ω. These results indicate that the thermal flow sensor is capable of measuring fluid flow in this channel with a lower detection limit of approximately 320 pL/s. The results in this experiment show a qualitative agreement with measurements shown in [13] even though the device and measurement configurations are substantially different. The PDMS film may be providing significantly higher thermal isolation allowing the measurement of fluid flow without pulsed mode techniques. However, pulsed mode operation will have an advantage of reduced sensitivity to ambient temperature fluctuations of the surrounding medium.

## 4. Conclusion

This paper presents a first demonstration of temperature control and fluid flow sensing in microchannels molded in polydimethylsiloxane (PDMS) using embedded IC-based microheating elements. The paper also describes a novel method to create a fluid interface to capillary tubing using commercially available capillary tubing connectors. Future work will be in optimization of the IC-based microheating elements, further thermal characterization, and application of the microheating elements for control of microchannel chemical reactions. The silicon machining can be performed from the backside of the chip so that microheating elements could be designed with openings on the topside; this would that allow solution to flow beneath the element. It is expected that optimization of the heater design will further increase the sensitivity of the sensor. In addition, since circuits can be monolithically integrated in this process, an improvement in the detection limit could be achieved by integration of a low-noise on-chip circuit and amplifier.

## Footnotes

1 Note: Silicon wafers can be purchased from the manufacturer with oxide predeposited.

2 It was noted that hillocks did form without the addition of the APODS, resulting in an extremely rough surface.

3 Certain commercial equipment, instruments, or materials are identified in this paper to foster understanding. Such identification does not imply recommendation or endorsement by NIST, nor does it imply that the materials or equipment identified are necessarily the best available for the purpose.

4 This assumes that the heating element is at the same temperature at each point on its surface where in actuality it is expected that there will be some distribution of temperature.
